# MicroRNA 21 and microRNA 10b: early diagnostic biomarkers of breast cancer in Egyptian females

**DOI:** 10.1186/s43046-022-00115-6

**Published:** 2022-04-11

**Authors:** Mai Ali, Dina El Gayar, Normeen Hany, Abdel Hamid Ezzat, Reham Zeyada

**Affiliations:** 1 Faculty of Medicine, Cairo University, Cairo, Egypt; 2 National Cancer Institute, Cairo, Egypt

**Keywords:** miR21, miR10b, Breast cancer, Noninvasive biomarkers, Early detection

## Abstract

**Background:**

Breast cancer (BC) is one of the most prevalent cancers in developing and developed countries among women worldwide. Mammography is one of the superior methods for BC detection, but it carries up to 20% false-negative results, especially in early cases. Histological examination of tissue biopsies and fine-needle aspiration cytology are invasive techniques. Hence, minimally invasive markers are needed for the improved detection of BC. microRNAs, small, noncoding, single-stranded RNAs functioning as tumor suppressor genes or oncogenes, are attractive biomarkers for early detection. This study aimed to examine the serum levels of miR21 and miR10b in patients with BC especially in the early stages compared to healthy controls to evaluate their potential use as BC biomarkers.

**Methods:**

This study included 90 females who were divided into two groups. Group I included 70 patients with BC and was subdivided into group Ia with 40 nonmetastatic BC patients and group Ib with 30 metastatic BC patients. Group II included 20 apparently healthy females as a control group. Serum miR21 and miR10b as biomarkers and *miR16* as a housekeeping gene were evaluated using real-time polymerase chain reaction.

**Results:**

The median levels of miR10b and miR21 were statistically significantly upregulated in the sera of patients with BC compared to healthy controls (*P* = 0.001). Receiver operating characteristic curve analyses demonstrated that serum levels of miR10b and miR21 were useful biomarkers for distinguishing between patients with BC and the control group, with an area under the curve (AUC) of 0.991 with 97.1% sensitivity and 100% specificity at a cutoff of 3.1 for miR10b and an AUC of 0.965 with 95.7% sensitivity and 85% specificity at a cutoff of 1.7 for miR21. Regarding the early stages of BC, the median levels of the fold change of serum miR21 and miR10b were statistically significantly higher in patients with BC (stages I and IIa) than in the control group (*P* < 0.001).

**Conclusions:**

Both miR21 and miR10b have valuable diagnostic roles in detecting the early stages of BC.

## Background

Breast cancer (BC) is universally the second most commonly diagnosed cancer that affects approximately 1 in 8 women during their lifetime [1]. Annually, 1.7 million women are diagnosed with BC, with a death toll amounting to 25% of all cancer cases and 14% of cancer-related deaths [2]. According to the National Cancer Institute statistics, among Egyptian women, it ranks first, accounting for 29% of cancer cases [3]. Globally, BC incidence varies dramatically, where the highest incidence is reported in developed countries, such as North America and Western Europe, with more than 90 new cases per 10^5^ women annually, remarkably higher compared to less than 30 per 10^5^ women annually in Eastern Asia [2] and 33.3 per 10^5^ women in Egypt (from 2009 to 2011) [4]. However, mortality rates have recently decreased due to improved screening and emerging adjuvant therapy [5].

In Egypt, a disturbing statistic is the age at diagnosis that is lower than in developed countries, such as North America and Europe, where most diagnosed females are premenopausal [3].

BC diagnosis relies on clinical examination with imaging and is confirmed by pathological assessment. Clinical examination includes bimanual palpation of the breasts and regional lymph nodes. Imaging includes the ultrasound of the breast and regional lymph nodes and bilateral mammography [6], which can, unfortunately, miss up to 20% of the early cases [7]. Consequently, for an accurate diagnosis, especially in those cases, BC diagnosis involves more invasive diagnostic measures, such as FNAC and histological examination of core biopsy [8].

However, besides the highly variable range of sensitivity and diagnostic accuracy of FNAC smears, depending on the experience of the cytopathologist, a major drawback is the inability to diagnose some benign or borderline breast lesions where core biopsy is superior [8]. Being both invasive procedures, there is a need to develop novel markers that are minimally invasive for the improved detection of BC.

MicroRNAs (miRNAs) are noncoding, small RNA molecules that affect gene expression via the suppression of translation or mRNA degradation [9]. miRNAs regulate crucial cellular processes, such as differentiation, proliferation, invasion, migration, and apoptosis [10]. The expression patterns of miRNAs are commonly changed in various pathological conditions, such as cancers [11]. Deregulation patterns of miRNAs are often observed in a wide range of cancers. Deregulation includes miRNAs that are overexpressed and hence considered oncogenes or oncomiRs, enhancing tumor occurrence, development, or metastasis. Other miRNAs originally used to inhibit oncogenic mRNAs are considered tumor suppressors in patients with cancer [12].

Furthermore, the regulation of these abnormal miRNAs can offer great scope for tailored therapies [13].

This study aimed to examine the serum levels of miR21 and miR10b in patients with BC compared to healthy females; assess their potential use as biomarkers for BC diagnosis, especially in the early stages; and correlate the serum levels with the clinicopathological features and various stages of the disease.

## Methods

### Study population

Blood samples were drawn from 70 patients with BC (group I) between October 2015 and December 2016. Group I was subdivided into two groups: group Ia with 40 nonmetastatic patients with BC and group Ib with 30 metastatic patients with BC. All patients enrolled in this work had a histologically confirmed BC diagnosis. Twenty serum samples were collected from healthy females serving as the control group (group II). All groups were age-matched, ranging from 25 to 70 years.

Sample size calculation was done using the G power program, and the recommended sample size for the current study is 20 in each group (20 patients in the metastatic group, 20 patients in the nonmetastatic group, 20 as the control group).

It was anticipated that a difference of 29 fold change exists between miR10b in patients compared to the controls and 16 fold change in miR21 at *P* value 0.01 and power of 95%.

The recommended number of individuals to be recruited was at least 40 patients (20 in each group). But it was decided to increase the sample size to 70 patients (40 in the nonmetastatic group and 30 in the metastatic group aiming at increasing the power of the study).

This study was approved by the Ethical Committee. Patients were screened for their eligibility to participate in the study. Informed consent was obtained from all eligible patients.

### Detection of miR21 and miR10b

#### Serum samples and preparation of total RNA

Serum samples were stored at − 80 °C until processing. RNA was isolated from the serum using the miRNeasy Mini Kit (Qiagen GmbH, Hilden, Germany) according to the manufacturer’s instructions. RNA concentration was determined by measuring the absorbance at 260 nm using the Quawell Q5000 UV-vis spectrophotometer.

### Reverse transcription (RT)

cDNA was reverse transcribed from total RNA samples using the miScript miRNA RT Kit (Qiagen) according to the manufacturer’s instructions. The RT master mix was prepared. Each RNA sample was mixed with 4 μL of 5× miScript HiSpec buffer, 2 μL of 10× nucleic mix, and 2 μL miScript reverse transcriptase mix. Template RNA and RNase-free water were added to each tube containing RT mix according to the RNA concentration to reach a final volume of 20 μL. The mixture was incubated at 37 °C for 60 min, 95 °C for 5 min, and then held at 4 °C in the Biometra thermal cycler.

Two mature miRNAs were detected (miR10b and miR21) using the SYBR Green polymerase chain reaction (PCR) miRNA primer assay. *miR16* was used for normalization. The sequences of mature miRNAs were identified using the miRbase.

### Real-time PCR (RT-PCR)

After RT, PCR products were amplified from cDNA samples with the SYBR Green PCR Kit (Qiagen). The cDNA product (5 μL), 2 μL miScript Specific Primer Assay (forward primer), 2 μL miScript Universal Primer (reverse primer), and 1 μL RNase-free water were mixed with 10 μL QuantiTect SYBR Green PCR Master Mix. The PCR conditions included an initial activation step at 95 °C for 15 min, followed by 40 cycles of 94 °C for 15 s, 55 °C for 30 s, and 70 °C for 30 s. The reaction was carried out in the StepOne RT-PCR system (Applied Biosystems, Foster City, CA, USA).

### Data analysis

#### Comparative cycle threshold (CT) method

The miRNA expression level was measured using the CT method. The expression for each miRNA was given by the difference between its CT value and the average CT value of the reference gene per sample [14]. The expression of circulating miR21 and miR10b was normalized through relative fold change (FC = 2^−∆∆CT^). *miR16* was used as a reference gene. It was expressed at high levels in the serum and calculated relative to its expression in the serum of age-matched healthy controls. Using *miR16* as a reference gene, the relative expression (FC) for each tested miRNA within each group was then calculated using the following equation: 2^−ΔΔCT^ [15].

#### Statistical analysis

Data were analyzed using IBM SPSS advanced statistics version 20 (SPSS, Inc., Chicago, IL, USA). Quantitative data were summarized as the mean ± standard deviation when normally distributed and median (25th–75th) when abnormally distributed. The relative expression levels of miR21 were characterized by their median and range from the 25th to 75th percentiles. Comparison of miR21 levels in the different patient clinical stages was analyzed using the Kruskal-Wallis test, and comparison of miR21 levels in hormone receptor status was analyzed using the Mann-Whitney test.

Qualitative data were expressed as the frequency and percentage. The chi-square test was used to examine the association between qualitative variables. For quantitative data not normally distributed, a comparison between the two groups was done using the Mann-Whitney test (nonparametric *t*-test).

Receiver operating characteristic (ROC) curves were constructed for different miRNAs, monitoring the area under the curve (AUC) and calculating the sensitivity, specificity, and accuracy at different cutoff levels. All tests were two-tailed. *P* < 0.05 was considered significant.

## Results

Table 1 summarizes the demographic, clinical, and pathological data of the BC groups (metastatic and nonmetastatic).

**Table 1:** Demographic, clinical, and pathological data of the BC groups (metastatic and nonmetastatic)

Parameters		Nonmetastatic group (***n*** = 40)	Metastatic group (***n*** = 30)	***P*** value
**Age groups**	**< 45 years**	14 (35%)	4 (13.3%)	
	**> 45 years**	26 (65%)	26 (86.7%)	
**Menopausal state**	**Pre**	18 (45%)	7 (23.3%)	0.063
	**Post**	22 (55%)	23 (76.7%)	
**Family history**	**Negative**	35 (87.5%)	26 (86.7%)	0.921
	**Positive**	5 (12.5%)	4 (13.3%)	
**Oral contraceptive pills**	**Negative**	24 (60%)	19 (63.3%)	0.780
	**Positive**	16 (40%)	11 (36.7%)	
**Tumor size**	**< 2**	31 (77.5%)	13 (43.3%)	0.003
	**> 2**	9 (22.5%)	17 (56.7%)	
**BC pathology**	**Duct**	34 (85%)	29 (96.7%)	0.108
	**Lobular**	6 (15%)	1 (3.3%)	
**BC subtype**	**Luminal A**	15 (39.5%)	15 (55.6%)	0.184
	**Luminal B**	12 (31.6%)	7 (25.9%)	0.606
	**Enriched**	8 (21.1%)	3 (11.1%)	0.271
	**Basal**	3 (7.9%)	2 (7.4%)	0.938
**Breast cancer grade**	**1**	0 (0%)	4 (13.3%)	0.018
	**2**	36 (90%)	22 (73.3%)	0.068
	**3**	4 (10%)	4 (13.3%)	0.669
**Lymph node**	**Negative**	14 (35%)	0 (0%)	0.0003
	**Positive**	26 (65%)	30 (100%)	
**T**	**T1**	2 (5%)	4 (13.3%)	0.222
	**T2**	31 (77.5%)	8 (26.7%)	< 0.0001
	**T3**	5 (12.5%)	7 (23.3%)	0.238
	**T4**	2 (5%)	11 (36.7%)	0.0008
**N**	**N0**	14 (35%)	0 (0%)	0.0003
	**N1**	24 (60%)	25 (83.3%)	0.036
	**N2**	2 (5%)	3 (10%)	0.424
	**N3**	0 (0%)	2 (6.7%)	0.099
**Estrogen receptor (ER)**	**Negative**	11 (27.5%)	6 (20%)	0.472
	**Positive**	29 (72.5%)	24 (80%)	0.658
**Progesterone receptor (PR)**	**Negative**	13 (32.5%)	8 (26.7%)	0.602
	**Positive**	27 (67.5%)	22 (73.3%)	
**HER2**	**Negative**	19 (47.5%)	25 (83.3%)	0.002
	**Positive**	21 (52.5%)	5 (16.7%)	
**Combined ER/PR**	**ER−/PR−**	11 (27.5%)	5 (16.7%)	0.290
	**ER+/PR−**	2 (5%)	3 (10%)	0.424
	**ER−/PR+**	0 (0%)	1 (3.3%)	0.250
	**ER+/PR+**	27 (67.5%)	21 (70%)	

The median values of fold changes of miRNAs in both studied groups are shown in Table 2, whereas the median values of fold changes of miRNAs in the nonmetastatic versus metastatic groups are shown in Table 3.

**Table 2:** The median values of fold changes (FC) of miRNAs in both studied groups

miRNAs fold change^**a**^ of the studied groups			
	BC (***n*** = 70)	Control (***n*** = 20)	***P***
**miR10b**	29.2 (12.1–73.4)	1.3 (0.8–2.01)	< 0.001
**miR21**	16.9 (7.0–29.7)	0.6 (0.2–1.2)	< 0.001

All data are presented as median (25th–75th percentiles)

The median values of FC are shown relative to reference miR-16

^a^Fold change formula: fold change = 2^−∆∆CT^

**Table 3:** The median values of fold changes (FC) of miRNAs in the nonmetastatic and metastatic groups

miRNAs fold change^a^ of the studied groups			
	Nonmetastatic group (***n*** = 40)	Metastatic group (***n*** = 30)	***P*** value
**miR10b**	31.6 (13.04–76)	26.1 (10.7–10.7)	0.529
**miR21**	18.5 (7.08–32.5)	13.3 (7.03–27.3)	0.198

^a^Fold change formula: fold-change = 2 ^-∆∆CT^

The ROC curve analyses for serum miR10b and mi R21 in patients with BC versus healthy controls are shown in Table 4.

**Table 4:** ROC curve analyses for the serum levels of miR10b and miR21 in patients with BC versus healthy controls

	AUC	***P***	95% CI		Cutoff	Sensitivity (%)	Specificity (%)	PPV	NPV	Accuracy
			Lower bound	Upper bound						
**FC of miR10b**	0.991	< 0.001	0.975	1.000	3.1545	97.1	100	100	90.9	97.7
**FC of miR21**	0.965	< 0.001	0.928	1.000	1.7582	95.7	85	95.7	85	93.3

*95% CI* confidence interval, *PPV* positive predictive value, *NPV* negative predictive value

The median values of fold changes of the studied miRNAs in the early stages of BC versus the control group are shown in Table 5.

**Table 5:** The median values of fold changes (FC) of the studied miRNAs regarding early stages of BC versus the control group

miRNA FC of the studied groups			
	Stages 1 and 2A (tumor size < 2 cm) (***n*** = 12)	Control group (***n*** = 20)	***P*** value
**miR10b**	21.1 (7.4–56.3)	1.3 (0.8–2.1)	< 0.001
**miR21**	14.2 (6.9–28.8)	0.5 (0.2–1.5)	< 0.001

The ROC curve analyses of serum miR10b and miR21 in the early stages of BC versus the control group are shown in Table 6.

**Table 6:** ROC curve analyses of the serum levels of miR10b and miR21 in the early stages of BC versus the control group

	AUC	***P*** value	95% CI		Cutoff	Sensitivity (%)	Specificity (%)	PPV	NPV	Accuracy
			Lower bound	Upper bound						
**FC of miR10b**	0.933	< 0.001	0.838	1.000	3.0609	91.7	95	91.7	95	93.8
**FC of miR21**	0.975	< 0.001	0.931	1.000	2.6065	100	90	85.7	100	93.8

Association of miR-10b and miR-21 and clinicopathological features in breast cancer patients are shown in Table 7.

**Table 7:** Association of miR-10b and miR-21 and clinicopathological features in breast cancer patients

Characteristics		miR-10b	miR-21
**Age groups**	**< 45 years (*n*= 18)**	42.24 (15.97–116.73)	22.96 (9.81–47.31)
	**> 45 years (*n*= 52)**	27.70 (11.33–56.38)	15.45 (6.75–24.34)
	***P*value**	**0.313**	**0.162**
**Menopausal state**	**Pre (*n*= 25)**	32.60 (14.49–78.63)	27.36 (15.71–37.90)
	**Post (*n*= 45)**	27.80 (11.37–57.96)	13.87 (4.27–21.02)
	***P*value**	**0.650**	**0.008**
**Family history**	**Negative (*n*= 61)**	30.63 (13.90–78.63)	18.05 (8.48–30.57)
	**Positive (*n*= 9)**	24.54 (11.29–51.88)	14.87 (4.27–17.08)
	***P*value**	**0.636**	**0.094**
**Oral contraceptive pills**	**Negative (*n*= 43)**	27.61 (10.76–53.71)	17.2 (4.36–35.36)
	**Positive (*n*= 27)**	34.23 (15.97–116.73)	15.93 (7.33–27.55)
	***P*value**	**0.252**	**0.819**
**Tumor size**	**< 2 cm (*n*= 44)**	29.22 (13.90–78.63)	19.75 (5.69–37.25)
	**> 2 cm (*n*= 26)**	29.12 (12.18–70.38)	15.56 (7.33–18.56)
	***P*value**	**0.927**	**0.079**
**Breast cancer pathology**	**Duct (*n*= 63)**	27.61 (12.18–70.38)	16.84 (6.47–27.74)
	**Lobular (*n*= 7)**	32.60 (6.48–178.16)	21.02 (15.18–78.47)
	***P*value**	**0.660**	**0.207**
**Breast cancer subtype**	**Luminal A (*n*= 30)**	38.53 (19.39–78.63)	17.68 (8.48–29.73)
	**Luminal B (*n*= 19)**	24.54 (11.37–78.63)	18.82 (12.76–42.05)
	**Enriched (*n*= 11)**	30.63 (10.76–70.38)	15.18 (2.63–32.76)
	**Basal (*n*= 5)**	14.49 (13.90–51.88)	7.33 (6.47–19.89)
	***P*value**	**0.761**	**0.29**
**Breast cancer stage**	**1 (*n*= 2)**	32.49 (8.21–56.77)	20.34 (2.78–37.90)
	**2A (*n*= 10)**	21.07 (6.62–55.99)	14.24 (9.81–19.89)
	**2B (*n*= 22)**	27.42 (13.90–78.63)	25.34 (14.87–47.31)
	**3A (*n*= 5)**	46.75 (37.19–158.35)	17.2 (4.36–18.05)
	**3B (*n*= 2)**	112.12 (30.63–193.61)	18.1 (15.18–21.02)
	**4 (*n*= 29)**	27.61 (10.76–57.96)	12.76 (7.03–25.33)
	***P*value**	**0.674**	**0.435**
**Breast cancer grade**	**Grade 1 (*n*= 4)**	100.34 (52.20–169.51)	13.10 (7.06–25.24)
	**Grade 2 (*n*= 58)**	25.90 (11.29–73.36)	18.30 (8.48–29.73)
	**Grade 3 (*n*= 8)**	29.38 (18.52–63.57)	9.90 (4.31–17.20)
	***P*value**	**0.128**	**0.305**
**Lymph node**	**Negative (*n*= 14)**	21.22 (8.21–56.77)	14.24 (3.98–21.02)
	**Positive (*n*= 56)**	30.63 (13.04–78.63)	17.14 (7.18–29.73)
	***P*value**	**0.618**	**0.725**
**T**	**T1 (*n*= 6)**	57.36 (46.43–142.72)	13.10 (2.78–36.61)
	**T2 (*n*= 39)**	24.20 (12.18–70.38)	21.47 (9.81–47.31)
	**T3 (*n*= 12)**	40.26 (14.47–155.66)	13.22 (6.12–18.30)
	**T4 (*n*= 13)**	24.54 (9.83–34.23)	12.76 (7.03–17.32)
	***P*value**	**0.315**	**0.153**
**N**	**N0 (*n*= 14)**	21.22 (8.21–56.77)	14.24 (3.98–21.02)
	**N1 (*n*= 49)**	30.63 (13.90–78.63)	17.32 (7.03–29.73)
	**N2 (*n*= 5)**	21.07 (11.29–46.75)	16.84 (11.27–19.62)
	**N3 (*n*= 2)**	23.17 (5.93–40.42)	10.62 (8.48–12.76)
	***P*value**	**0.735**	**0.791**
**M**	**M0 (*n*= 41)**	31.62 (13.04–76.0)	18.56 (7.08–32.55)
	**M1 (*n*= 29)**	26.07 (10.76–57.96)	13.32 (7.03–27.36)
	***P*value**	**0.529**	**0.198**
**Estrogen receptor (ER)**	**Negative (*n*= 17)**	30.63 (13.90–51.88)	7.33 (2.63–19.89)
	**Positive (*n*= 53)**	27.80 (12.18–78.63)	18.05 (10.51–29.73)
	***P*value**	**0.758**	**0.066**
**Progesterone receptor (PR)**	**Negative (*n*= 21)**	14.49 (9.56–46.75)	7.33 (2.636–19.89)
	**Positive (*n*=49)**	35.43 (15.97–78.63)	18.05 (11.27–29.73)
	***P*value**	**0.102**	**0.032**
**HER2**	**Negative (*n*= 44)**	27.70 (11.01–68.30)	17.26 (7.18–27.46)
	**Positive (*n*= 26)**	30.63 (14.49–73.36)	15.45 (4.36–32.76)
	***P*value**	**0.794**	**0.841**
**Combined ER/PR**	**ER−/PR−**	27.00 (12.33–61.33)	11.25 (2.72–26.33)
	**ER+/PR−**	9.56 (8.21–9.83)	2.78 (2.35–18.56)
	**ER−/PR+**	35.43 (35.43–35.43)	1.45 (1.45–1.45)
	**ER+/PR+**	35.71 (15.97–78.63)	18.30 (11.80–30.15)
	***P*value**	**0.122**	**0.034**
**Metastatic work up**	**Free (*n*= 40)**	31.62 (13.04**–**76.00)	18.56 (7.08**–**32.55)
	**Bone (*n*= 10)**	52.20 (27.80**–**142.72)	13.32 (11.27**–**30.57)
	**Liver (*n*= 6)**	31.35 (9.30**–**78.63)	9.90 (7.33**–**18.82)
	**Bone, liver (*n*= 5)**	24.54 (15.97**–**34.23)	15.93 (7.03**–**17.08)
	**Bone, lung (*n*= 6)**	10.29 (6.95**–**14.49)	21.10 (2.35**–**32.76)
	**Liver, lung (*n*= 1)**	35.43 (35.43**–**35.43)	1.45 (1.45**–**1.45)
	**Bone, lung, liver (*n*= 2)**	19.05 (13.90**–**24.20)	16.92 (6.47**–**27.36)
	***P*value**	**0.194**	**0.583**

Data are presented median (25th**–**75th percentiles)

**Fig. 1:**
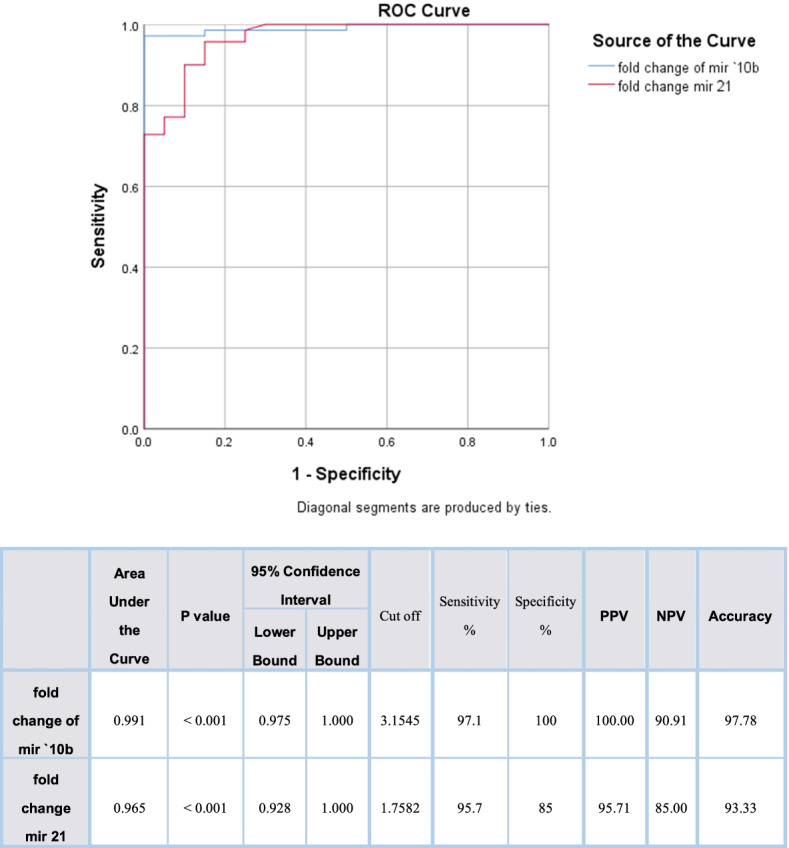
ROC curve analyses showing the AUC of the studied miRNAs in BC versus the control group

**Fig. 2:**
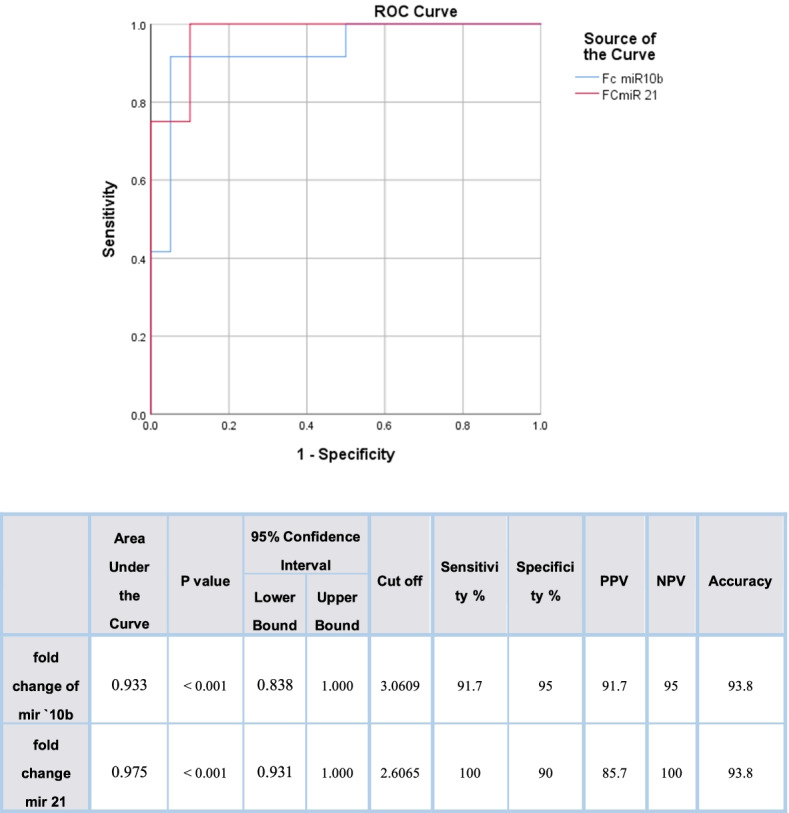
ROC curve analyses showing the AUC of the studied miRNAs in early-stage BC versus the control group

The ROC curve analyses showing the AUC of the studied miRNAs in patients with BC versus the control group are shown in Fig. 1, whereas the ROC curve analyses showing the AUC of the studied miRNAs in early-stage patients with BC versus the control group are shown in Fig. 2.

## Discussion

In this study, the serum levels of miR21 and miR10b were significantly upregulated in patients with BC with a median fold change (16.9 and 29.2, respectively) compared to healthy controls with a median fold change (0.6 and 1.3, respectively) (*P* = 0.001).

In agreement with these results, previous studies [16–19] have found that the serum level of miR21 was significantly upregulated in patients with BC compared to healthy controls (*P* = 0.0001).

ROC curve analysis was done to assess the validity of serum miR21 and miR10b as diagnostic markers for BC. In this study, miR10b and miR21 have demonstrated a strong discriminating ability between patients with BC and healthy controls. ROC curve analysis demonstrated an AUC of 0.965, sensitivity of 95.7%, and specificity of 85%, at a cutoff value of 1.7 for miR21 and an AUC of 0.991, sensitivity of 97%, and specificity of 100% at a cutoff value of 3.1 for miR10b. Therefore, miR21 and miR10b can be useful biomarkers for distinguishing between healthy controls and patients with BC. However, both markers could not discriminate between various stages of BC or between metastatic and nonmetastatic groups.

The focus of this work was a marker for the early detection of BC, as this would offer patients a significantly higher survival rate. When BC is detected in the early stages (stages I and IIA), the survival rate is 87%, whereas it drastically drops to 13% in stage IV with late detection*.* Mammography in the early stages in tumors < 2 cm has a sensitivity of 88% and a specificity of 93% [20], whereas core biopsy and FNAC have a sensitivity of 97% and specificity of 92%; however, both require expertise and are invasive procedures [8].

The latest guidelines [21] for screening patients with BC aimed to detect cancer in the early stages, so it is recommended to have annual mammography done. Furthermore, women at high risk of BC (positive family history and BRCA mutations) would benefit from mammography and annual screening with magnetic resonance imaging.

The median levels of serum miR21 and miR10b in patients with BC stages (I and IIA) (14.2 and 21.1, respectively) were statistically significantly higher compared to healthy controls (0.5 and 1.3, respectively; *P* < 0.001).

ROC analyses were plotted to assess the sensitivity, specificity, and diagnostic accuracy of these markers, which were found to be superior in the early detection of BC. miR21 at a cutoff value of 2.6 showed a sensitivity of 100%, specificity of 90%, and diagnostic accuracy of 93%. miR10b at a cutoff value of 3.1 showed a sensitivity of 91.7%, specificity of 95%, and diagnostic accuracy of 93%. Those findings highlighted their role as useful early diagnostic biomarkers for distinguishing early-stage patients with BC from healthy controls.

Positive HER2 expression was found to promote tumor aggression, invasion, and progression [22]. The relationship between miR21 and HER2 was investigated in this work in several clinicopathologic aspects. There was a significant finding only regarding menopausal status. The serum level of miR21 was statistically significantly higher in premenopausal patients with BC than postmenopausal patients (*P* = 0.008). Similarly, the incidence of HER2-positive patients was higher in the premenopausal group compared to the postmenopausal group (14/25 versus 12/45; *P* = 0.02).

However, there was no statistically significant difference in the expression level of miR21 regarding HER2 status (*P* = 0.841). This finding was similar to a previous study [16, 20]. Therefore, miR21 might be independent of HER2 status.

Regarding PR status, the miR21 expression level was statistically significantly higher in PR-positive than PR-negative patients (*P* = 0.018). Also, the miR21 level was statistically significantly higher in double-positive hormone receptor patients (ER+/PR+) than other combined hormonal receptor patients (ER+/PR−, ER−/PR+, and ER−/PR−; *P* = 0.034). These results were similar to a study done by [22] that found miR21 level was statistically significantly higher in PR-positive than in PR-negative patients (*P* = 0.018) and statistically significantly higher in certain combined hormonal receptor status groups (ER+/PR+) than in the combined hormonal receptor (ER+/PR−, ER−/PR+, and ER−/PR−; *P* = 0.006).

The prognosis of patients with BC and response to endocrine therapies are highly influenced by ER and PR expression levels. Unfortunately, some patients become resistant to standard therapies. That is why it is important to add additional factors, such as miR21. This will not only be valuable for patient stratification but also in searching for new kinds of therapeutics, such as anti-miR therapy [22].

The results showed no statistically significant difference in the expression level of miR21 or miR10b regarding ER status (*P* = 0.066 and 0.758). Similarly, there was no statistically significant difference in the expression level of miR21 or miR10b regarding BC grade or pathology (duct and lobular; *P* = 0.305, 0.128, 0.207, and 0.660, respectively) and different clinical stages and BC subtypes (luminal A, luminal B, basal, and enriched; *P* = 0.435, 0.674, 0.29, and 0.761, respectively). No statistically significant difference in the expression level of miR21 or miR10b in patients with BC regarding lymph node metastasis (*P* = 0.725 and 0.618, respectively) or different metastatic sites (bone, brain, lungs, and liver; *P* = 0.583 and 0.194, respectively) was found.

The limitation of our study is although this is the recommended sample size however bigger sample size would be more informative if more patients in the early stage were included.

## Conclusion

As mentioned previously, early detection offers a survival rate that vastly surpasses that with late detection. In this study, miR21 and miR10b not only discriminated patients with BC from healthy controls but were also found to be valuable diagnostics tools in the early stages with a sensitivity of 100% and 91.7%, specificity of 90% and 95%, and diagnostic accuracy of 93% and 97%, respectively, compared to mammography (sensitivity of 88% and specificity of 90%) and FNAC and core biopsy (sensitivity of 97% and specificity of 92%).

## Data Availability

The data that support the findings of this study are available on request from the corresponding author

## Abbreviations

BC: Breast cancer
FNAC: Fine-needle aspiration cytology
miRNA: MicroRNA
PCR: Polymerase chain reaction
FC: Fold change
PR: Progesterone receptor
ER: Estrogen receptor
HER2: Human epidermal growth factor receptor 2
ROC: Receiver operating characteristic
AUC: Area under the curve
CI: Confidence interval
PPV: Positive predictive value
NPV: Negative predictive value
